# Case Report: Missed diagnosis of twin reversed arterial perfusion sequence discovered at delivery: lessons from poorly monitored monochorionic twin pregnancy

**DOI:** 10.3389/fmed.2026.1812519

**Published:** 2026-05-28

**Authors:** Hanane Houmaid, Myriem Sali, Karam Harou, Bouchra Fakhir, Lahcen Boukhanni, Hamid Asmouki, Abderraouf Soummani

**Affiliations:** Gynecology Obstetrics Department, Mohammed VI University Hospital, Cadi Ayyad University, Marrakesh, Morocco

**Keywords:** acardiac acephalus fetus, case report, monochorionic twin pregnancy, twin reversed arterial perfusion, ultrasonography

## Abstract

Twin reversed arterial perfusion (TRAP) sequence is an extremely rare and serious complication that occurs specifically in multiple monozygotic pregnancies. Early sonography and Doppler examination are the cornerstones for an accurate diagnosis. This is a case of an acardiac acephalus twin who was misdiagnosed during pregnancy and discovered at vaginal birth after 36 weeks of gestation in a 31-year-old woman, gravida three para two. The pregnancy was poorly followed up and was mistakenly considered a singleton. It is a rare complication that affects monochorionic multiple pregnancies and is the best explanation for the associated morphological abnormalities. Diagnosis can be made through ultrasound examination at an earlier stage of pregnancy, enabling effective monitoring of the healthy twin who is at the risk of heart failure and anemia. There are many therapeutic methods aimed at preserving the healthy fetus from complications by suppressing the vascularization of the acardiac fetus. This case highlights a rare and serious complication of monochorionic twin pregnancy and emphasizes the need for multidisciplinary care. Radiologists and obstetricians should be attentive to pregnant women who have an abnormal tissue mass alongside a normal twin without any cardiac activity. An ultrasound scan is essential for the accurate diagnosis of the TRAP sequence; an early diagnosis and treatment can help save the pump twin and increase its chances of survival without cardiac complications.

## 1 Introduction

The twin reversed arterial perfusion (TRAP) sequence, also known as acardiac twin or chorioangiopagus parasiticus, is an extremely rare congenital malformation that occurs uniquely in multiple monozygotic pregnancies (1, 2). The incidence is estimated at 2.6% of monochorionic twin pregnancies and 1 in 9,500 to 11,000 of all pregnancies (3).

In recent years, this incidence appears to have increased due to improved antenatal diagnosis, the widespread use of ultrasound, and the rising prevalence of *in vitro* fertilization (IVF) (4). The pathogenesis of the TRAP sequence remains unclear; however, two hypotheses have been proposed: abnormality in placental vascularization or a defect in cardiac embryogenesis (5). This condition is characterized by the presence of a twin that appears as a tissue mass without an identifiable heart (or with only a rudimentary heart), often lacking upper limbs. Consequently, it is described as a severely malformed or anomalous fetus (6).

This acardiac twin is entirely dependent on the blood circulation of the normally developed co-twin (the pump twin) via arterio-arterial or arterio-venous placental anastomosis. Consequently, the pump twin faces an increased risk of cardiac overload, heart failure, and polyhydramnios, leading to a perinatal mortality rate up to 55% (7).

The primary objective is to interrupt blood flow to the acardiac twin in order to ensure the continued development and wellbeing of the pump twin (8).

We report a rare case of the TRAP sequence that was discovered only at the time of delivery after the pregnancy had been misidentified as a singleton. We also discuss the diagnostic pitfalls and practical insights for an early ultrasound assessment.

This case report has been prepared in accordance with the CARE guidelines, and written informed consent was obtained from the patient for publication.

## 2 Case presentation

### 2.1 Patient information

A 31-year-old pregnant woman (gravida 3, para 2) was referred from a peripheral hospital while in active labor. Her obstetric history included one prior uncomplicated term vaginal delivery 4 years earlier and one spontaneous miscarriage 2 years prior. She had no relevant medical history, no consanguinity, and no known hereditary disease. Antenatal follow-up was insufficient, and gestational age was estimated at 36 weeks based on a single first-trimester ultrasound performed at 7 weeks’ gestation by a general practitioner. No abnormalities were reported, chorionicity was not assessed, and multiple pregnancies were not identified. No further imaging, laboratory investigations, or specialist consultations were conducted; consequently, the pregnancy was mistakenly identified as a singleton gestation.

### 2.2 Clinical finding

Upon admission, the patient was hemodynamically stable with a blood pressure level of 130/70 mmHg and a temperature of 37 °C. The obstetric examination revealed a fundal height of 28 cm and an externalized breech presentation at the vulvar level, along with ruptured membranes and an absence of detectable fetal heart sounds.

### 2.3 Timeline

At 7 weeks of gestation, a first ultrasound was performed, which reported a singleton pregnancy without assessing chorionicity.

During pregnancy, antenatal follow-up was inadequate, with no additional imaging or investigations.

At 36 weeks of gestation, the patient was admitted in active labor.

Upon admission, intrauterine fetal demise was suspected in a presumed singleton pregnancy.

However, during delivery, a malformed fetus was followed by the successful birth of a healthy newborn.

The postpartum examination confirmed a monochorionic diamniotic twin pregnancy complicated by the TRAP sequence.

### 2.4 Diagnostic assessment

A diagnosis of intrauterine fetal demise (IUFD) in a presumed singleton pregnancy was made on admission.

Since the delivery was imminent, no intrapartum ultrasound examination was performed.

**Figure 1:**
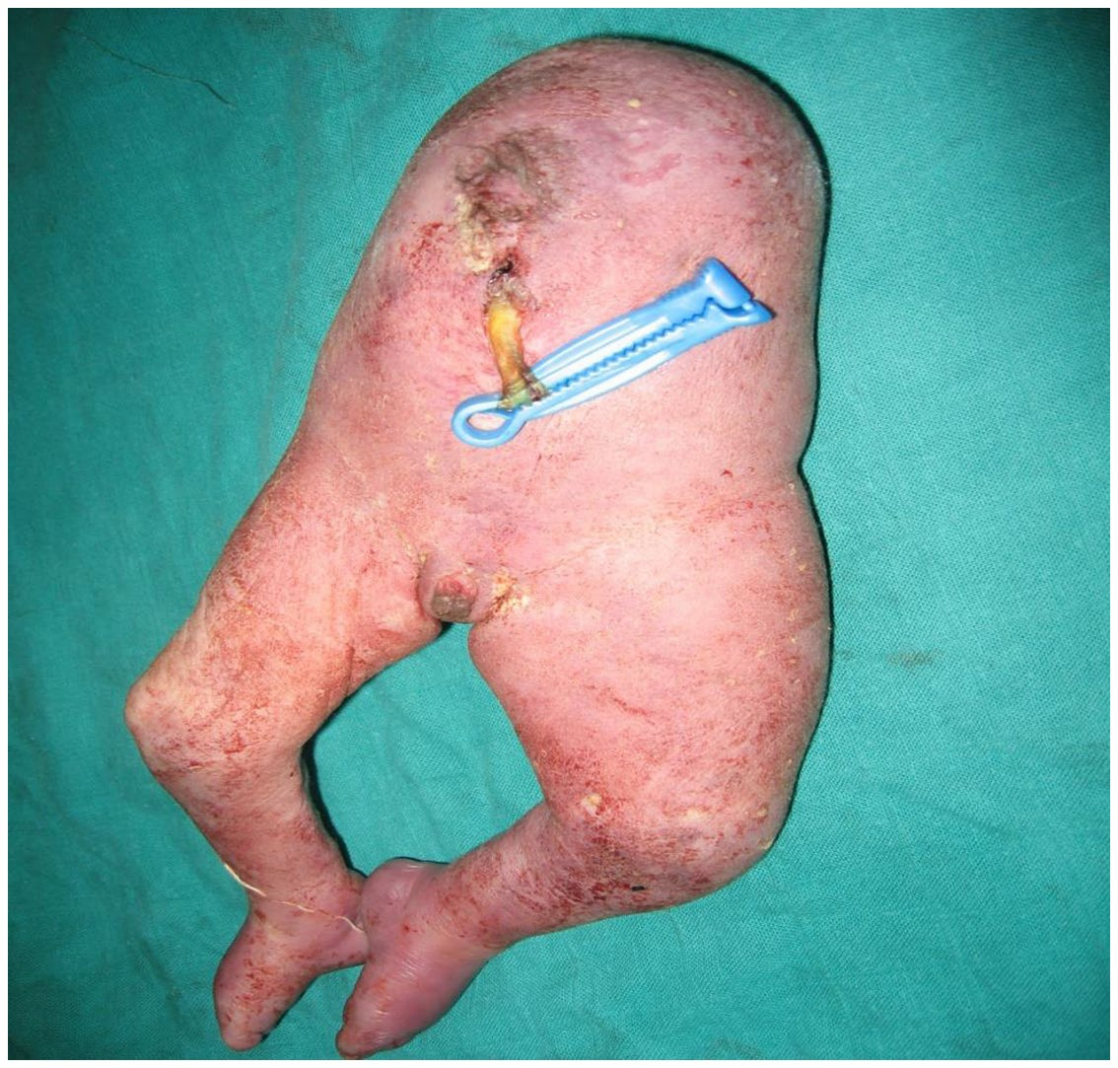
Acephalic acardiac fetus.

The patient delivered a severely malformed female fetus that showed no cardiac activity, lacked upper limbs, has absent thoracic structures, and was missing a cephalic pole (Figure 1).

**Figure 2:**
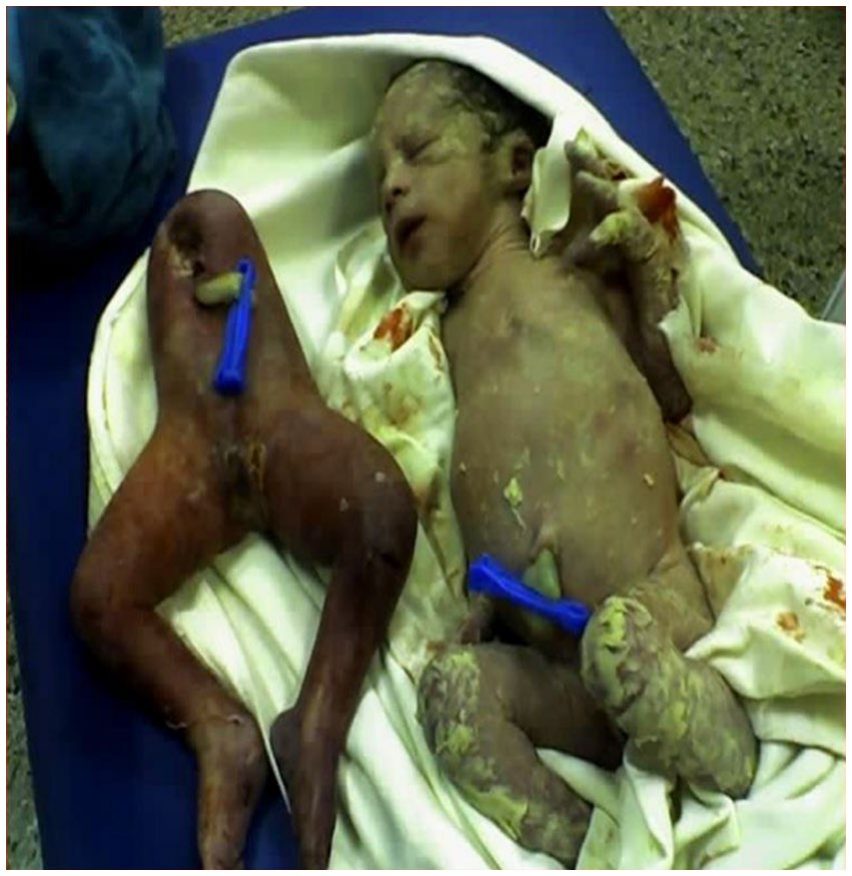
Acardiac twin and pump twin immediately after vaginal delivery.

After the initial delivery, persistent uterine contractions followed, with a second amniotic sac protruding into the vagina. An artificial rupture of membranes revealed another breech presentation, and a second female newborn was delivered vaginally without complications, with Apgar scores of 8/10 at 1 min and 10/10 at 5 min, a birth weight of 2,200 g, and normal external morphology (Figure 2).

The macroscopic examination of the placenta revealed a single placental mass with two unequal amniotic cavities, consistent with monochorionic diamniotic twinning.

The umbilical cord of the pump twin contained three vessels (two arteries and one vein), whereas the acardiac twin had a two-vessel cord consisting of a single umbilical artery and one enlarged vein.

**Figure 3:**
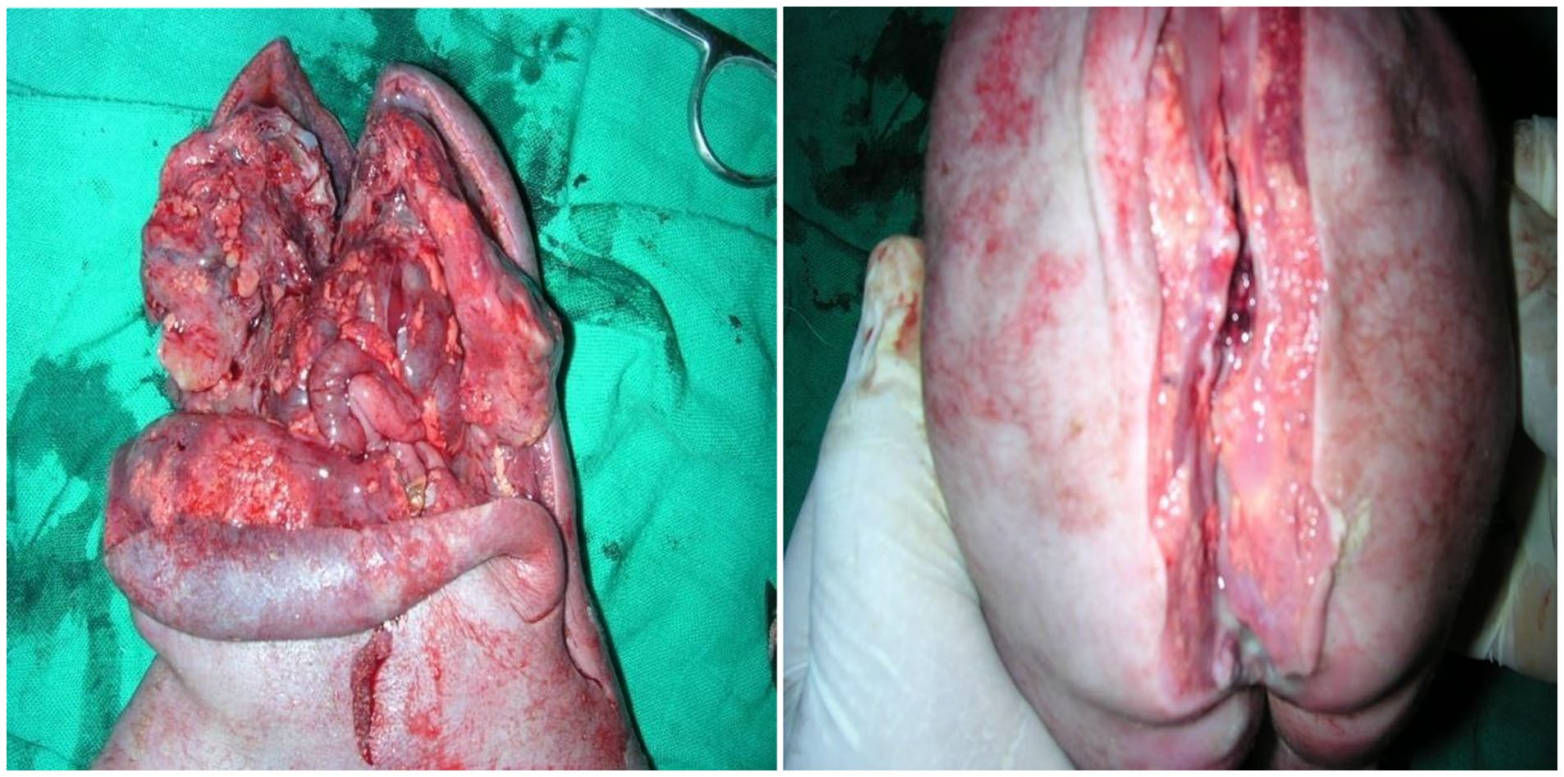
Postmortem examination of the acardiac twin.

The postmortem examination of the malformed fetus revealed a female fetus weighing 1,000 g and measuring 20 cm in length. The findings included an avascular tissue mass with no identifiable cardiac structures, an absence of head and upper body structures with only a few hairs observed at the upper pole. The abdomen and lower limbs were present, with four toes on each foot. There were no identifiable thoracic or abdominal organs. A short segment of the intestine ended in a blind pouch, and the external genitalia appeared normal (Figure 3).

Plain radiography revealed the pelvis and long bones of the thighs, legs, and foot phalanges. The overall findings confirmed an acardiac acephalus fetus in a previously unrecognized monochorionic diamniotic twin pregnancy complicated by the twin reversed arterial perfusion sequence. The postpartum maternal course was uneventful, and no maternal complications were recorded.

### 2.5 Therapeutic intervention

No antenatal therapeutic intervention was performed due to a missed diagnosis during pregnancy. The patient had a vaginal delivery.

### 2.6 Follow-up and outcomes

The second twin (known as the pump twin) was delivered vaginally without complications, with Apgar scores of 8/10 at 1 min and 10/10 at 5 min. The birth weight was 2,200 g, and the external morphology was normal.

The neonate remained clinically stable; however, cardiac auscultation revealed a ventricular septal defect, and the newborn was admitted to the neonatal intensive care unit for monitoring and specialized management, with no signs of heart failure or neonatal anemia.

The acardiac twin was non-viable.

The maternal postpartum course was uneventful, and no maternal complications were recorded.

## 3 Discussion

Twin pregnancy refers to the simultaneous development of two fetuses within the uterine cavity and can be either dizygotic or monozygotic. Approximately 20% of twin pregnancies are monochorionic (MC) (9). Twin pregnancies, particularly monochorionic ones, are associated with higher perinatal mortality and morbidity compared to singleton pregnancies. Second-trimester miscarriage rates are approximately 6% for monochorionic twins compared to 1.9% for dichorionic (DC) twins. Stillbirth rates range from 4 to 6% in monochorionic twins, while these rates are just over 1% in dichorionic twins and approximately 0.5% in singleton pregnancies (10).

Adverse outcomes in twin pregnancies are primarily related to prematurity and complications specific to multiple gestations, including conjoined twins, acardiac twins, congenital malformations, hypertensive disorders, gestational diabetes, fetal growth restriction, intrauterine fetal demise, and neonatal death (11–13).

The twin reversed arterial perfusion (TRAP) sequence occurs exclusively in monochorionic multifetal pregnancies, as placental vascular anastomoses are essential for its development. Monochorionic placentation additionally carries risks related to vascular communications within the placenta, which may lead to twin-to-twin transfusion syndrome (TTTS) and twin anemia-polycythemia sequence. These complications affect approximately 10–15% of monochorionic twin pregnancies (13).

Although the TRAP sequence is most commonly described in twin gestations, rare cases in triplet pregnancies have also been reported (1–12). It represents a severe form of twin vascular pathology, affecting approximately 1% of monochorionic twin pregnancies. Approximately 75% of cases occur in diamniotic twins and 25% in monoamniotic twins. Abnormal arterio-arterial placental anastomosis accounts for the specific complications observed (13–16).

In the TRAP sequence, the acardiac twin receives deoxygenated arterial blood from the pump twin through arterio-arterial anastomoses. Consequently, the upper body of the acardiac twin often develops poorly, resulting in a severely dysmorphic fetus. The pump twin supports both circulations and is therefore at a high risk of cardiovascular complications, including congestive heart failure, hydrops fetalis, intrauterine fetal demise, polyhydramnios, preterm labor, and premature delivery (2, 13–19). Perinatal mortality of the pump twin has been reported to range from 35 to 55% (17). The TRAP sequence can be classified based on cardiac development into the following types:

- Holocardius: complete absence of cardiac tissue.
- Hemicardius: presence of a rudimentary heart.

A morphological classification is widely used and primarily descriptive. Although it is older and lacks a therapeutic orientation or prognosis interest, it remains common due to the fact that the acardiac fetus is non-viable in all cases (8–23).

1. Acardius acephalus: This is the most common type, characterized by the absence of the head, thoracic organs, and upper limbs, while the pelvis and lower limbs are relatively preserved.
2. Acardius anceps: This is the most developed type, with a partially formed head and facial structures and relatively well-developed limbs.
3. Acardius acormus: This type is rare, characterized by the presence of cephalic structures only.
4. Acardius amorphous: This type of acardiac twin has a shapeless mass of tissue without identifiable anatomical structures.

Our case corresponds to the acardius acephalus type.

Umbilical cord abnormalities occur in up to 97% of acardiac twins, with a single umbilical artery being the most frequent anomaly (24). Chromosomal abnormalities have been reported in approximately 50% of acardiac twins and 9% of pump twins (21, 25), highlighting the potential value of genetic evaluation.

Antenatal diagnosis of the TRAP sequence relies primarily on ultrasound examination. Early detection during the first trimester is possible, and biometric discordance is often an early indicator (26). Cardiac activity may initially be observed in the acardiac twin but subsequently disappear, supporting the vascular theory of dysmorphogenesis (27). Movements of the acardiac twin are typically passive and reflect the activity of the pump twin. Differential diagnoses include vanishing twin syndrome, placental teratoma, anencephaly, and intrauterine fetal demise (28).

Doppler examination shows abnormally high resistance in the umbilical artery of the acardiac twin, and reverse arterial flow strongly supports the diagnosis (7). The difference in the resistive index between the twins appears to correlate with the pregnancy outcome (29). In the pump twin, ultrasound monitoring should assess signs of cardiac failure such as pleural effusion, ascites, hepatosplenomegaly, polyhydramnios, skin edema, and hydrops fetalis.

The acardiac fetus is non-viable and represents a lethal complication that threatens the life of the pump twin. The pump twin faces a high risk of intrauterine death, either due to cardiac failure from hemodynamic overload or prematurity secondary to polyhydramnios. The prognosis primarily depends on the acardiac-to-pump twin weight ratio and Doppler indices of the pump twin’s umbilical artery (30).

The management strategy aims to protect the pump twin by interrupting vascular connections. Expectant management, which involves close ultrasound monitoring, may be appropriate for small acardiac twins that show no signs of cardiac failure (30, 31). In cases of rapid growth or large acardiac mass, invasive interventions are considered, including radiofrequency ablation (31, 33), cord coagulation (32, 35), or intrafetal vessel occlusion (7, 34, 35). Radiofrequency ablation (RFA) is currently the most widely used and least invasive technique.

Our case illustrates the consequences of inadequate prenatal monitoring and an inability to determine chorionicity early in pregnancy due to operator inexperience. The pregnancy was misclassified as a singleton due to limited follow-up and the absence of systematic ultrasound assessment. This prevented early diagnosis and appropriate monitoring. Early diagnosis of the condition could have allowed referral to a specialized fetal medicine unit, Doppler surveillance, and consideration of fetal interventions to improve the outcome of the pump twin.

This case highlights important clinical lessons: chorionicity should be systematically assessed and documented during first-trimester ultrasound. Any abnormal tissue mass adjacent to a normal fetus should prompt targeted Doppler evaluation. In addition, discordant fetal morphology should raise concerns about monochorionic complications and require referral to specialized care.

### 3.1 Patient perspective

The patient reported that her pregnancy follow-up was limited and she was unaware that she was carrying twins. She expressed surprise upon receiving the diagnosis during delivery but felt reassured by the positive outcome of the healthy newborn. She recognized the importance of regular antenatal care and early ultrasound evaluation.

### 3.2 Strengths and limitations

This case describes a rare and severe complication of monochorionic twin pregnancy with detailed clinical, placental, and postnatal findings, allowing accurate diagnosis of the TRAP sequence. It also highlights the important diagnostic pitfalls related to inadequate antenatal surveillance and failure to assess chorionicity, providing valuable educational insights, particularly in low-resource settings. However, the lack of adequate prenatal follow-up and imaging limited early diagnosis and prevented the assessment of the condition’s progression and the consideration of fetal intervention. In addition, genetic investigations were not conducted, which could have provided valuable information given the reported association between the TRAP sequence and chromosomal abnormalities.

## Data Availability

The original contributions presented in the study are included in the article/supplementary material, further inquiries can be directed to the corresponding author/s.
